# External ballistics of Pleistocene hand-thrown spears: experimental performance data and implications for human evolution

**DOI:** 10.1038/s41598-018-37904-w

**Published:** 2019-01-25

**Authors:** Annemieke Milks, David Parker, Matt Pope

**Affiliations:** 1 Institute of Archaeology, University College London, 31-34 Gordon Square, London, WC1H OPY UK; 2 Nordic Sport (UK) Limited, 21 Bentley Road, Castle Donington, Derby, DE74 2UL UK

## Abstract

The appearance of weaponry - technology designed to kill - is a critical but poorly established threshold in human evolution. It is an important behavioural marker representing evolutionary changes in ecology, cognition, language and social behaviours. While the earliest weapons are often considered to be hand-held and consequently short-ranged, the subsequent appearance of distance weapons is a crucial development. Projectiles are seen as an improvement over contact weapons, and are considered by some to have originated only with our own species in the Middle Stone Age and Upper Palaeolithic. Despite the importance of distance weapons in the emergence of full behavioral modernity, systematic experimentation using trained throwers to evaluate the ballistics of thrown spears during flight and at impact is lacking. This paper addresses this by presenting results from a trial of trained javelin athletes, providing new estimates for key performance parameters. Overlaps in distances and impact energies between hand-thrown spears and spearthrowers are evidenced, and skill emerges as a significant factor in successful use. The results show that distance hunting was likely within the repertoire of hunting strategies of Neanderthals, and the resulting behavioural flexibility closely mirrors that of our own species.

## Introduction

The use of hand-thrown spears emerged some time during the Pleistocene, and is thought to have been an important step in human evolution, functioning not only as a precursor to mechanically-projected weaponry, but also as a weapon used for hunting and interpersonal violence until the ethnographic present. In general early weaponry is used as a proxy for a wide range of behaviours including hunting and scavenging^1–5^, self-defence against dangerous animals^6^, human-human violence^7^, social behaviours and group size in relation to cooperative hunting^8–10^, the development and use of language and teaching^10,11^, cognitive abilities necessary for selection of raw materials, design, and use^12–16^, and human dispersal events and species replacement^7,17^. According to models of early weaponry, hand-thrown spears chronologically follow the development of thrusting spears, but precede the development of mechanically-aided ‘complex’ projectiles such as spearthrowers and bow/arrows, which are sometimes called ‘true’ projectiles^17–21^. Similarly, it is argued that the ability to ‘kill at a distance’ is not present until the advent of complex projectiles^17,22,23^.

Although hand-thrown spears have an important role in human evolution, their performance and effectiveness as hunting weapons remains poorly understood.

According to multiple sources^17,21,22,24–31^ Pleistocene hand-thrown spears have numerous disadvantages in relation to subsequent innovations. Characterisations include that they are heavy, which makes them difficult to throw and achieve high release velocities, with further loss of velocity in flight; that they are less lethal than complex projectiles due to lower levels of kinetic energy (KE) at impact; that there are limitations on the distance from which they can be deployed compared with complex projectiles, and are inaccurate, which in turn increases danger and discoverability; and therefore there are limitations on hunting strategies and prey for hominins using hand-thrown spears. Many of these theories are based on selective or unquantified data, resulting in a lack of clarity about which aspects were likely to be true limitations^4^. Spear throwing experiments typically involve the use of inexperienced throwers and/or replicas designed for other delivery systems^25,32,33^. Data from studies involving experienced throwers typically relates to studies launching lighter objects such as throwing sticks which have different ballistic properties^34^.

Evidence-based debates around the origins of thrusting and throwing spear use in human evolution have typically focused on hominin skeletal evidence. Proposals that features of the upper limbs of different species of *Homo* indicate that throwing only comes into play with *H*. *sapiens*^23,35^ are hampered by multiple issues. These include small sample sizes, human variation in populations^36^, evidence that humeral robusticity and shape may not correlate with strains in weapon use^37^, and a lack of clarity whether any single activity contributes to or offsets bone remodeling or robusticity^36,38,39^. Others argue for an earlier emergence of throwing, showing that features necessary for accurate and powerful throwing are evidenced in *H*. *erectus* fossils^40–42^. A recent find of an early Neanderthal dating to MIS 7 from Tourville-la-Rivière shows skeletal trauma consistent with repeated throwing, supporting the hypothesis that they were capable and frequent throwers^43^.

Archaeology can and should make a valuable contribution to the debate on spear throwing but thus far systematic research into the ballistics of early spears as thrown weapons has lagged behind both palaeoanthropological research and archaeological approaches to complex projectiles, although such research is beginning to be undertaken^33,44^. The earliest spear is a fragment that dates to ca. 400,000 BP from Clacton-on-Sea (UK), and was crafted out of yew^1,45^. The site of Schöningen (Germany) dates to ca. 300,000 BP and has yielded at least 10 complete and nearly complete wooden spears, most of which are made of slow-growing spruce^46,47^. Most of the wooden spears from Schöningen are tapered at both ends^47^ and of those whose maximum diameter locations have been published, the widest diameters are located in the front half^46^ suggesting that the hominins manufacturing them may have intentionally designed at least some of the spears as flight weapons for hunting (See Supplementary Information regarding design requirements for flight). Evaluating the ballistics of thrown spears is valuable not only for the analysis of specific archaeological sites and artefacts, but also for broad chrono-geographical comparisons of hunting behaviours throughout the Eurasian Palaeolithic, African Stone Age, Australian archaeological contexts, and Paleoindian archaeological record, all of which have suitable if rare examples of fragments or complete one-piece untipped pointed artefacts interpreted as spears^45–52^, as well as lithic and osseous points proposed to be the hafted components of hand-delivered spears^16,26,33,53–57^. Continued use of thrown spears among recent or extant foragers and pastoralists is well-evidenced^22,58–60^, suggesting this weapon played an important role throughout the Pleistocene and Holocene.

Current knowledge of the performance parameters of hand-thrown spears, including effective distance, impact velocities, kinetic energy (KE), momentum and flight behaviour rests primarily upon estimates or data that are not directly relatable (Supplementary Information Table S1), and/or selected ethnographic reviews^22,58,59^. The most influential of the ethnographic reviews proposed that the effective distance of thrown spears is limited to 5–10 m^22^. This estimate is on the basis of a mean of accuracy estimates from the ethnographic literature and has not adequately accounted for the significance of throwing skills amongst groups such as the Tiwi and Tasmanians who did not use complex projectiles. This distance estimate has been challenged elsewhere citing throwing distances of heavy javelins by Roman soldiers^61,62^. We add further data here that are question the proposed limit of 10 m. The Tiwi threw very heavy spears with accuracy according to one source being up to 50 m (Supplementary Information Table S4) even if they typically chose to approach prey closely when possible, as any hunter would. The Tasmanians threw lighter spears with accuracy distances reportedly exceeding 50 m (Supplementary Information Table S4). Additional examples of accuracy distances well exceeding 5–10 m exist including from groups in mainland Australia, Africa, and Papua New Guinea (see Supplementary Information Section 1.2 and Table S4). Including these data in an analysis would increase accuracy distance estimates on the basis of ethnographic use by highly skilled throwers to at least 15–20 m, if not well beyond. However we reject the utility of a mean in this particular case because such a comparison brings together groups who threw spears regularly with those who threw rarely, whose spears were very different in mass and design, who threw spears for different purposes, and who hunted prey with different body sizes and behaviours in a variety of environments.

Velocity data related to the hand-throwing of Pleistocene spears by humans captured with high speed video (HSV) or other precision recording equipment are rare (Supplementary Information Table S1). Replication studies that mechanically fire prehistoric weapons rely on estimates in order to evaluate fracture patterns, microwear analysis, and wound ballistics (Supplementary Information Table S2). Problems with existing velocity and therefore kinetic energy (KE) and momentum (*p*) estimates are multiple and include the use of unskilled throwers, the lack of accurate recording equipment, and inappropriate replicas such as hand-throwing of spearthrower darts. Velocities captured with appropriate recording equipment of skilled throwers - such as those from studies of Olympic javelin athletes - relate to release not impact (Supplementary Information Table S1), with the relationship between these variables in field conditions poorly understood^63^. In general, estimates regarding the use of prehistoric weaponry have relied upon both foundational and ongoing ethnoarchaeological work, with multiple studies using human participants in naturalistic settings contributing to performance data of complex projectiles. Furthermore the recreational and professional use of spearthrowers and bow/arrows is currently widespread while there are few who habitually throw weapons by hand in similar contexts, creating a present-day skill gap between hand-delivered weapons and complex projectiles.

A clearer understanding of release and impact velocities, KE, momentum and throwing distance aids in evaluating the Pleistocene archaeological record, and more generally facilitates an evidence-based assessment of current estimates around hand-thrown spear performance. Understanding flight paths and impact angles contributes to our ability to design replicative experiments, evaluate potential bone lesions to prey resulting from hominin hunting activities^64^, and assess human lesions potentially occurring from violent encounters^7^. This paper provides the first systematic data addressing the external ballistics of hand-thrown spears, with external ballistics (or exterior ballistics) defined as the ‘branch of applied physics which deals with the actions and characteristics of missiles or projectiles in flight’^65^. It also deals with terminal ballistics, or ‘the branch of applied physics which deals with the actions and characteristics of missiles or projectiles as they approach the target’^66^. The results contribute important data for those investigating wound ballistics, which concerns the morphology and size of wounds. Known principles and physics of the ballistics of hand-thrown spears and javelins are outlined in the Supplementary Information and elsewhere^31,67–72^.

## Results

A throwing trial captured data on the external ballistics of replicas of Schöningen Spear II using six male athletes trained in the javelin throw. A total of 120 throws took place with each participant performing 20 throws. One hundred and two throws were aimed at a target at a series of distances and 12 were for maximum distance.

### Results: Velocity

**Figure 1:**
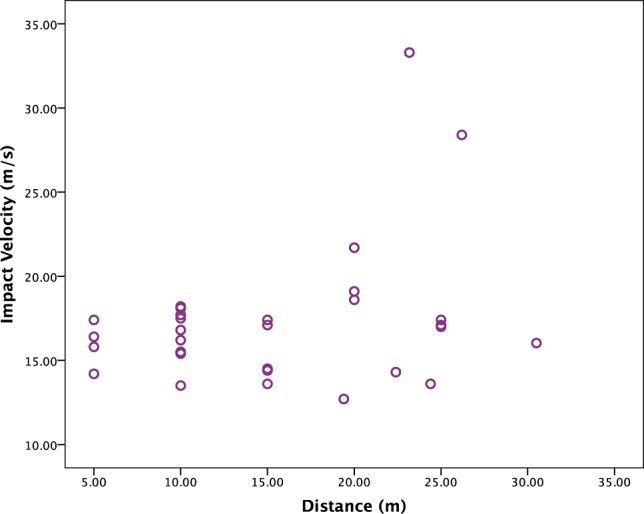
Scatterplot of impact velocities by distance.

In total thirty-six throws, or recorded events (REs), were captured on HSV. Release velocities ranged from 11.8–20.11 m/s (Table 1). Impact velocities for aimed throws ranged from 13.5–21.7 m/s. Impact velocities analysed by distance for all throws show overall an even distribution across the distances (Fig. 1). However, those at the 20 m distance are high in relation to those at other distances, and two outliers also occur at longer distances. Of the distance throws that could be analysed, impact velocities ranged from 12.7–33.3 m/s, with the highest velocity relating to a distance throw of 23.2 m, and the spear landing point first. Impact velocity data show variability by participant (Supplementary Information Fig. 6). For example, the participant with the least number of years’ experience (P5) also produced the lowest impact velocities. The two participants (P1 and P4) with the highest percentage of ‘hits’ (Supplementary Information Table S3) also produced the highest impact velocities.

**Table 1:** Descriptive statistics for velocity, kinetic energy and momentum, separated by data for aimed throws vs. those for all throws combined, including distance throws.

	Mean	Median	SD	Min	Max	n
**Aimed throws**						
Release velocity (m/s)	15.85	15.80	2.087	11.8	20.11	19
Impact velocity (m/s)	16.60	16.4	2.023	13.5	21.7	21
Rate of change in velocity (m/s)	0.29	0.50	1.044	−1.5	1.9	17
Kinetic energy (J)	107.6	104.4	26.47	71	179	21
Momentum (kg*m/s)	12.80	12.92	1.566	10	17	21
**All throws combined**						
Impact velocity (m/s)	17.26	16.80	4.15	12.70	33.30	31
Kinetic energy (J)	122.50	109.99	75.276	65	444	31
Momentum (kg*m/s)	13.42	12.93	3.368	10	27	31

**Figure 2:**
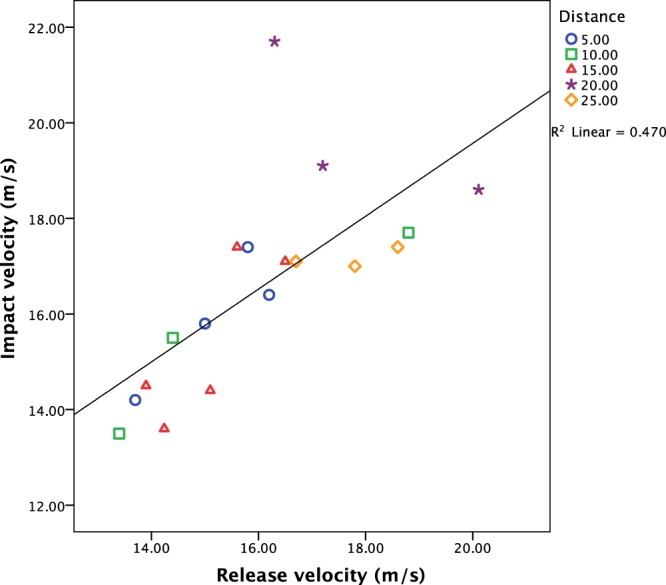
Regression analysis for release and impact velocity data.

A regression analysis of the 18 RE for which there are both release and impact velocities showed a moderate correlation (R^2^ = 0.470) between the two (Fig. 2). The relationship is stronger for slower velocities but is weakest for the 20 m distance.

**Figure 3:**
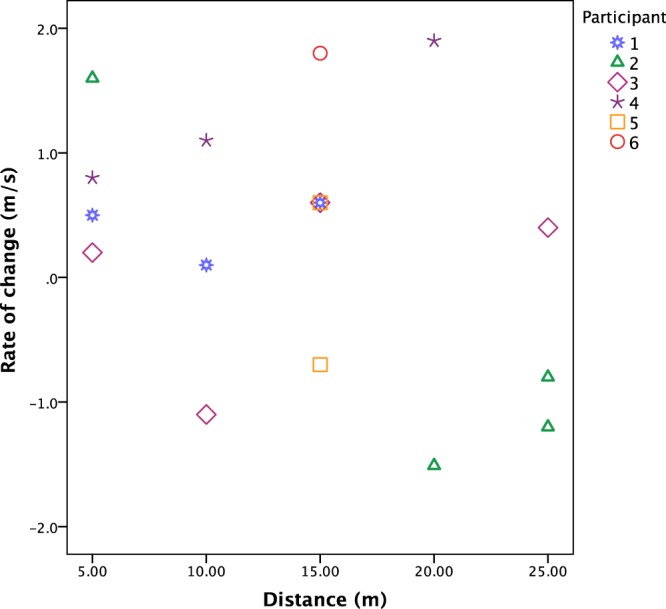
Rate of change (in m/s) by distance and participant.

Removing an outlier with a large increase, the remaining 17 REs demonstrate a trend for an overall slight increase in velocity from the point of release to the point of impact (Fig. 3). The rate of change from release to impact velocities ranged from −1.51 m/s to 1.84 m/s (Table 1). Both slight decreases and increases occurred across the range of distances, with the largest increase occurring at a distance of 20 m (Fig. 3). Gravitational acceleration (9.81 m/s^2^) in part explains the increases with acceleration from point of release to impact. Some velocity is gained through gravitational acceleration for all throws including parabolic trajectories because the release height of the spears was >1.5 m while impact velocity was measured at or near ground level (see Supplementary videos 2 and 4, and Supplementary Information for detailed explanation and equations). Spears thrown in a parabolic trajectory are affected by multiple forces during flight, and tail winds such as those experienced during this experiment can also increase velocity by increasing the horizontal velocity as well as affecting lift (see Supplementary Information Section 1.3 and Supplementary Information Fig. 1)^72,73^.

### Results: Kinetic Energy and momentum

The KE from the trial ranges from 71 to 179 J (Table 1) for aimed throws.

The mean KE of aimed throws (107.6 J) exceeds the maximum of complex projectiles due to the mass of the spears (Supplementary Information Table S3; Supplementary Information Fig. 5) further underscoring the significance of mass for hand-delivered weaponry. The range for all combined throws in this study is greater than for aimed throws, from 65 to 444 J. Although many of the distance impacts did not fall point first, the highest value of 444 J related to a point-first impact (RE 28). Momentum follows much the same pattern, ranging from 10–17 kg*m/s for aimed throws, with the lowest value for hand-thrown spears exceeding even the highest values for complex projectiles (Supplementary Information Table S3).

### Results: Distance and accuracy

**Figure 4:**
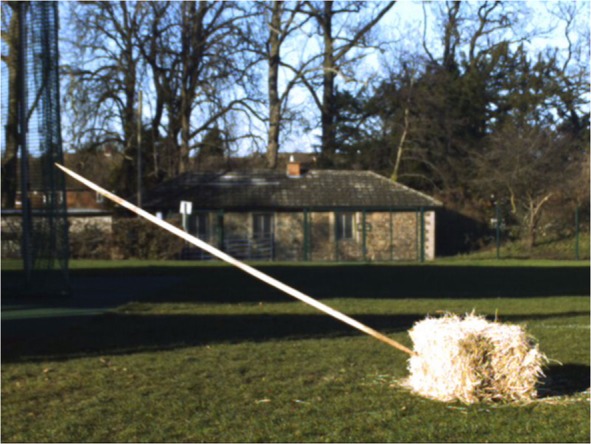
Still frame from high speed video footage showing a target hit.

**Figure 5:**
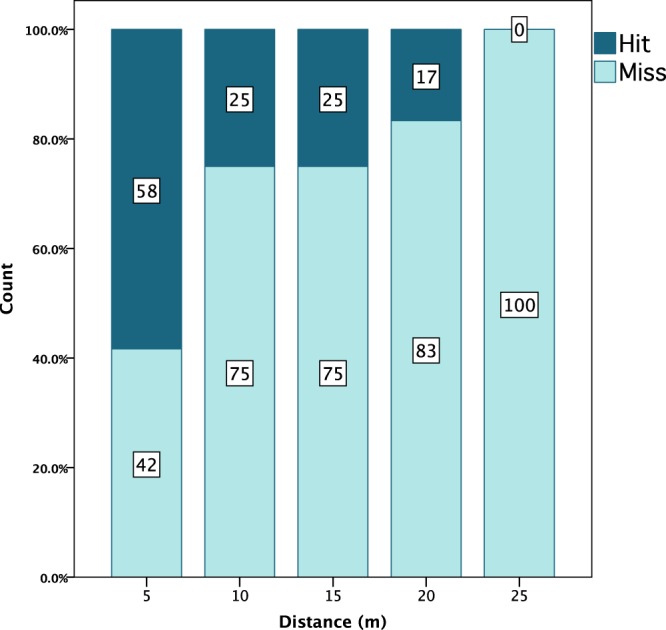
Hits and misses per distance expressed as percentages of total number of throws. 10 m distance includes all throws at this distance, including those with altered target height.

Of the 102 aimed throws, 25 hit the target (25%) (Fig. 4) while 77 (75%) missed entirely. A breakdown of the hit/miss data by distance confirms the correlation between target distance and accuracy (Table 2). When combining the data of all throws at 10 m, the percentage of hits follows a clear descending pattern by distance (Fig. 5). The additional hits performed at 10 m with increase in target height target greatly increased the number of hits: 17% of throws were hits at 10 m with the hay bale horizontal on the ground, while 33% resulted in hits when vertical, which is an increase of 16%. Hit rates at other distances may have been similarly improved with a higher target, meaning taller or standing prey may be easier to hit than prey closer to the ground. Participants showed variability in their ability to hit the target. The percentage of total hits by participant ranges from 11% to 33% (Supplementary Information Table S7). The participant with the fewest years’ throwing experience (P5) had the lowest percentage of hits, and the lowest impact velocities underscoring the significance of training. Participants with 5 years experience had the best hit rate. However, body mass also appears to have a role (Supplementary Information Fig. 7), with a relatively strong correlation between body mass and successful hit rate. Participant height did not have a clear correlation. Distance throws ranged from 19.4 m to 31.2 m (mean = 26.33 m; SD = 3.981 m). The most experienced participant (P2) who is accustomed to stiff distance-rated javelins achieved the farthest distance, while the one with least experience (P5) performed the poorest.

**Table 2:** Hit and Miss data by distance.

Distance (m)	Hits	Misses	% Hits	n
5	7	5	58	12
10*	9	27	25	36
15	6	18	25	24
20	3	15	17	18
25	0	18	0	18

^*^Including throws with vertical hay bale.

### Results: Flight trajectories

Throughout the experiment, two types of flight trajectories were observed. Flight paths for throws at 5 m had a downward-directed flat flight trajectory. Participants adjusted the angle of release according to each distance, with a maximum upward angle of release for the farthest distances (Supplementary Videos 1 and 2). At 5 m distance throws, the angle of release and impact fall within –0.20 and –0.40 radians (rad), corresponding to between −11° and −23° (Supplementary Information Figs 11 and 12). At 10 m release angles were near to zero, with impact angles being negative, beginning an increasing trend towards parabolic throws. From 10 m and beyond angles increase upwards in relation to the ground at release, and decrease by impact. These results demonstrate a correlation between release and impact angles and reflect the shift from flat flight trajectories at the closest distances to parabolic trajectories at the farther distances. The maximum release angle is 0.55 rad, which is a 35° angle, corresponding to a 25 m throw. Impact angles ranged from −0.57 to −0.22 rad, translating to impacts between −33° and −13°.

Spears oscillated (vibrated) along the long axis of the spear in flight and flexed upon impact (Supplementary Videos 3 and 4). Spears also spun in flight (Supplementary Video 4), a result of the throwers intentionally imparting spin, which improves stability^73^. Of the 25 suitable target impact videos (including both hits and misses), 84% (n = 21) landed point first, 12% (n = 3) landed rear first and 4% (n = 1) landed flat. In distances 20 m and beyond, both for target and distance throws, several examples of the spear yawing and stalling in flight were observed. For the distance throws, spears rarely landed point first (n = 3), typically landing with the proximal (back) of the spear first, which are failed landings. Yawing and stalling are influenced by release conditions, spear design, and conditions during flight including both wind and the orientation of the spear in relation to the flight trajectory, and it is likely that a combination of these factors contributed to these suboptimal flight behaviours (see Supplementary Information).

## Discussion

The mean impact velocity in this experiment provides data for replication studies, and while it largely confirms existing estimates of prehistoric spear throwing^25,74^, the spear replicas used in this study were of a higher mass, demonstrating that in skilled hands, such masses are not a limiting factor. According to the principle that the greater the mass of an object, the greater the work to propel that mass^75^, objects with lighter mass, such as those thrown by inexperienced throwers used for the calculations elsewhere^25^ would be expected to be thrown with significantly higher velocities by skilled throwers than those achieved in the present study using heavier spears. This is supported by the higher release velocities achieved with lighter thrown objects such as balls and sticks by skilled throwers (Supplementary Information Table S1). The range of velocities in the present study overlaps with velocities recorded for spearthrowers. The lack of a trend to drop in impact velocity from release differs from lighter and fletched projectiles such as arrows and darts, which experience more drag than a heavier unfletched spear (see Supplementary Information for further explanation). Overall the data from this experiment demonstrate that at short ranges in field conditions there is not a demonstrable loss in velocity and therefore KE or momentum from release to impact for spears such as those from Schöningen, and therefore the hypothesis that this is a significant limitation of the weapon is not supported. With different wind conditions and/or at greater distance throws there may be more significant velocity losses, though a recent javelin study also shows that loss in velocity from release to impact over longer distances can also be slight^76^. Further experimentation on this question would be useful.

Hand-thrown spears can impact with KE and momentum values that are suggested to be sufficient to hunt large prey^77^. Considering that it is impact energy, not just velocity, that influences penetration and fracture mechanics, these results are significant for those seeking to identify prehistoric weaponry delivery systems in the Pleistocene archaeological record using systematic mechanical projection. In addition to KE and momentum, other variables such as tip material and morphology affect penetration, influencing ability to cut through hide, muscle and bone, depth of penetration, and wound morphology. The spears used in this experiment have wide diameters in comparison with most complex projectiles, and do not have a stone tip. This study focuses on the external and terminal ballistics of the spears and does not address penetration and wounding capabilities, or overall effectiveness of these weapons, something that requires systematic experimentation.

The participants in this study, trained in throwing but not in aiming for a target, were able to hit the target at distances up to 20 m, though with a significant drop in accuracy at each distance. Their inability to hit a target at distances >20 m likely relates in part to their inexperience with target throwing, but additional factors most likely include the difficulty of aiming with a parabolic trajectory and the target height. It has been suggested that hitting a target with complex projectiles is a relatively easy skill to learn in comparison with other hunting skills^77,78^. This may or may not hold true for hand-thrown spears, which appear to require not only greater power to launch but are potentially more difficult to learn to reliably hit a target than spearthrower darts and arrows. The ethnographic evidence presented in this paper suggests that these skills can be learned for throwing, but that it may require years of regular practice to master, in turn affecting the number of hunters that a society may be able to deploy.

However, with practice in aiming improvement over the results in this experiment can be expected. Within our experiment, the participant with the best hit rate (33%) performed three times better than that with the worst hit rate (11%) (Supplementary Information Table S7). On the other hand, P2 with the most years throwing experience did not have the best hit rate (Supplementary Information Table S7), and thus other factors such as overall fitness and regular practice likely play a role. Body mass may also play a role in successful hit rates (Supplementary Information Fig. 7). Further supporting the importance of skill is the fact that our participants had an 11.4% improvement at the 15 m distance (25% hit rate) over the untrained participants in an experiment using the same size target, and replicas of the same spear (13.6% hit rate)^28^. Capturing data from groups trained in aimed throws, such as those skilled in use of the Roman pilae or extant hunter-gatherers, is a necessary step-wise experiment that will build upon current understanding of hand-delivered spears including whether skill, fitness or body mass play the most important role in accuracy, distance, and impact energy. We propose on the basis of ethnographic estimates and experimental data in this paper a conservative revision of distance estimates for hand-thrown spears of 15–20 m. This still likely represents a lower accuracy distance than complex projectiles, but the extent of the difference is not as great as is often suggested. The proposed revised estimate of 15–20 m is not intended to be definitive but rather to re-open the debate on this important question and invite further experimental use by trained throwers, and will take further naturalistic use of hand-thrown spears to better understand the true limitations of this delivery system.

On the basis of the experiment presented in this paper, two different throwing trajectories can be used. Flat trajectories at a target are an effective technique for shorter distances. At distances ≥15 m, hunters would need to use a parabolic trajectory, which decreases accuracy and requires more skill and strength. Distance throws were shown in this study to achieve significant impact KE and momentum, but frequently failed to land point first. Further studies will be necessary to understand whether this is a limitation of spears or due to the inexperience of most of our participants with stiffer javelins, typically used by elite throwing athletes (see Supplementary Information). This could in part explain why some throws experienced yawing as greater power is needed to accurately throw a stiffer spear. Spears such as those from Schöningen, manufactured from dense wood and with thick diameters, could also have been designed to match experienced and powerful throwers, balancing durability with aerodynamics, reflecting an understanding of material properties by Middle Pleistocene *Homo*.

The primary purpose of this paper was to replace problematic estimates of the external and terminal ballistics of hand-thrown spears by reviewing the existing evidence and collecting empirical data. The data support hypotheses that early spears, such as the double-tapered examples from Schöningen, function as throwing weapons both for flat and parabolic trajectories at distances up to 20 m. Our results underscore the importance of using trained participants if launching weapons manually in experimental research^79^ whether the aim be to further evaluate performance parameters or for replication work. In experimental work using human throwers, recording of impact velocities, accuracy data, and physical attributes and skill level of throwers will help fill in knowledge gaps. Unlike for spear thrusting, controlled mechanical replication of hand-thrown velocities is straightforward, but experimenters should carefully consider potential masses of hand-thrown spears as this affects external, terminal and wound ballistics. Replicating a mean statistic for weapon performance parameters while ignoring the range creates problems for comparing and identifying delivery systems. In particular replicating mean impact velocity and by extension KE and momentum rather than the range superficially suppresses human and technological variability, masks overlaps with other weapon systems, and may create false taxonomies.

The results imply that robust, highly-trained and habitual throwers could throw spears with more power and at least twice as far as has been widely argued for in the literature. The extension to a 20 m accuracy limit from this experiment implies greater flexibility in hunting strategies than the previous distance estimates. The larger ethnographic distances discussed would put hand-thrown spears on par with spearthrowers. In hunter-gatherer societies, the skills involved in hunting take decades to master, beginning in early childhood^78^. With both skill, strength and potentially a larger body size needed to use spears effectively as thrown weapons, we can theorize that only hominins that combined relatively large brains with robust physiques could have employed them as part of a successful subsistence strategy. These effective costs are high when compared to later complex projectiles that may have offered a relaxation on time and energy budgets required to learn and effectively use these ‘simple’ hunting technologies. The importance of skill in weapon use has been discussed elsewhere^31,70,80^, with an interesting hypothesis proposing that hand-delivered weapons are not inferior in terms of energy, but rather that part of their disadvantage relates to a high investment in training^31^. Understanding and explaining the development and persistence of hand-thrown weapons in the human past is complex and requires approaches from multiple disciplines. It is not the case that hand-thrown spears are universally replaced by complex projectiles, as they continued to be used, most typically alongside other weapon systems, through to the ethnographic present. Moreover we can compare this performance data with those from experiments demonstrating the potential of these weapons as thrusting spears, in order to consider the likelihood that they were routinely thrown at all. Understanding the fine detail of the situations, ecologies, and strategies in which hand-thrown spears were considered preferable to more complex weapons may now provide a useful avenue for understanding the context in which these weapons emerged in the first place.

## Methods

### Methods: Setup and data acquisition

**Figure 6:**
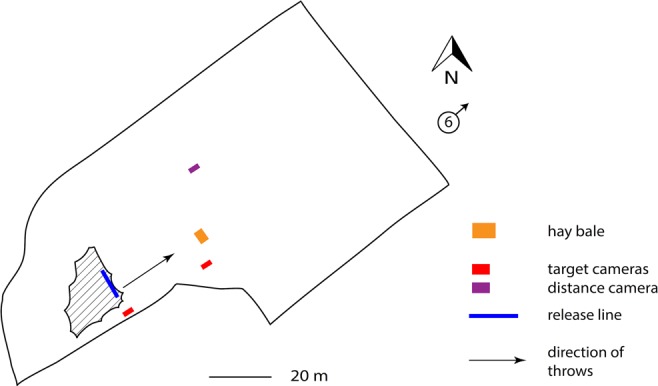
Map of Steve Backley National Throws Centre, where the experiment took place. The hashed area is the covered throwing area. The blue line is the release line. The red blocks show the positions of the Fastec cameras for target throws, and the purple block shows the location of the Fastec camera for the distance throws. The camera by the release line remained constant, while the camera positioned by the target (orange block) to capture impacts moved along with the target. The circle with the ‘6’ and arrow shows the direction of the wind in relation to the throwing field. The ‘6’ represents m/s and represents a sample wind speed, which varied between 5.3 and 8.9 m/s throughout the experiment. Drawn by A. Milks.

The experiment took place at the outdoor Steve Backley National Throws Centre, Loughborough University (Fig. 6). Participants were located under a covered area, with the throwing field uncovered. The mean air temperature was 3 °C, precipitation was 0.0 mm and wind speed was between 5.3 and 8.9 m/s (19–32 km/h) with no gusts, in a southwesterly direction (weather data accessed via Weather Underground website https://www.wunderground.com/). Participants threw towards the northeast, in the direction of the wind, thus the wind provided a tailwind for the throws (Fig. 6).

Throws included those at a target set at a series of distances, as well as throws to achieve maximum distance. One hundred and twenty throws were undertaken. Release throws and impacts were filmed using two HSV cameras (Fastec TS3 TS3100-S) with resolution at 800 × 600 at 1000 fps, determined by test trial, with significantly higher frame rates than studies capturing javelin and spear velocities (cf. Table S1). The release velocity camera was set at a right angle 8 meters from the release line, and the impact velocity camera for aimed throws was set at a right angle to the approaching spear trajectory, 8 metres from the centre of the face of the target. Both cameras were leveled. This setup facilitated capturing the nature of impact of the spear with the target, showing a view of the entire spear at impact, rather than just the tip of the spear at impact. The distance throw camera was set at a greater distance, allowing for a wider field of view, as distance throws had a wider landing range. HSV captured 92% of the target impacts. Four videos were retained for misses at longer distances to understand velocities at these distances. Release velocities could not be captured for maximum distance throws (n = 12) due to low light. The camera for distance throws was positioned farther away than cameras for the target shots in order to widen the field of view (Fig. 6). Digital photographs and standard video recordings (n = 14) were also made periodically throughout the experiment to capture examples of throwing techniques and flight trajectories.

### Methods: Spear replicas

Schöningen Spear II was chosen as the template for spear replicas because it had published measurement data and a scaled distal tip photograph available from which to replicate the dimensions, it represents a complete example, and is closest to mean values of the published measurement data from Schöningen available at the time of the experiment^2,81,82^. Spear replicas were crafted from Norwegian spruce (*Picea abies*) trees grown on limestone/clay in Kent (UK). Replicas were made by hand using metal tools, and the surface was worked at the final stage with lithic tools, creating a surface that accurately replicating the surface of a Pleistocene wooden spear. Two replicas were used, weighing 760 g and 800 g (Supplementary Information Table S5), which conform well to the mean mass of ethnographic hand-delivered wooden spears (701 g; n = 58)^60^.

### Methods: Target

A hay bale (105 cm × 55 cm × 50 cm) was placed horizontally on the ground at a series of distances (every 5 metres from 5 to 25 m) from a throwing line for the aimed throws. A series of additional throws (n = 18) took place at 10 m with the hay bale turned vertically to assess whether the height of the target affected accuracy. A ‘hit’ was defined as the spear making contact with the target, and therefore does not exclusively relate to penetrating impacts. This study was designed to understand human performance and spear ballistics, not to evaluate the effectiveness of these spears on an animal target or the damage to wooden spears. The trial therefore did not require a complex target, and a soft target was chosen to protect spear replicas from damage. The ‘kill zone’ or primary target area on an ungulate is relatively small^30^ although hits to other areas can also be lethal. Therefore, a hay bale of this size represented a target similar in size to the target area of an adult size class III ungulate^83^. This also provided a useful comparison with a previous informal throwing accuracy test^28^ that used replicas of the same spear and a hay bale for a target.

### Methods: Participants

Six males trained in the javelin throw aged 18 to 34 years old took part (Participants 1–6 = P1-P6) (Supplementary Information Table S6). Ethical approval from UCL was waived on the basis that no data were collected covertly, the data were fully anonymised and participants were not vulnerable. Participants were orally briefed, provided signed informed consent, and were aware they could withdraw at any stage of the work without penalty, and personal data was collected and stored in accordance with UCL regulations and policy. Any participant with identifiable information or images in videos or photographs is contained in the manuscript has given consent for this to be published in an online open access publication. Participants volunteered to take part in the throwing trial, and were not paid. Males were chosen to provide a homogenous sample, and because they achieve around 30% greater distances in their throws due primarily to higher release velocities^84,85^. All participants have received formal coaching in the javelin throw, varying from one to 24 years (mean = 8; SD = 8.3). Self-reported heights ranged from 1.73–1.89 m (mean = 1.82) and body masses ranged from 65–93 kg (mean = 81.7). Middle Pleistocene (MP) *Homo* manufactured the original Schöningen spear replicated for this study, and the height and body mass of the participants correspond well with estimates for MP males^86–90^. No coaching occurred on the day and athletes rotated to combat fatigue.

Participants were not permitted a run-up for aimed throws, but up to three steps were allowed to facilitate body position at release. For distance throws a small approach of up to five metres was permitted, though not always used, to facilitate the throwing action. Run-ups for the javelin throw are stipulated as being a minimum of 30 m^91^. A typical run up of ca. 30 m adds an average of 2.8 m/s for novice throwers and 4 m/s for ‘club’ level throwers (defined as a PB of >50 m) at the most to a release velocity^92^. Most (n = 4) of our throwers would qualify as ‘club’ throwers, with a few (n = 2) as novice. As the participants in this study only used a ≤5 m approach for the distance throws we estimate that those throws using a small run-up (P1, P3, P4, P5, P6) would have added ca. 0.5 m/s added release velocity over those (P2) who threw their distance throw from standing.

### Methods: Data analysis

The software package Phantom Cine Viewer v2.5.744.0 was used for HSV analysis to measure velocity, and angles of release and impact. Standard and HSV were also analysed for spear flight paths and spear performance to assess flight paths, flight behaviour and landing. Details of the HSV analysis are in the Supplementary Information and an example analysis can be seen in Supplementary Video 5.

## Supplementary information

Video 1

Video 2

Video 3

Video 4

Video 5

Supplementary Information

Supplementary Dataset

## Data Availability

Data generated from this study are included in this published article (and its Supplementary Information files). The original videos generated during the experiment are not publicly available due to the necessity to protect the anonymity of human participants but are available from the corresponding author on reasonable request.
