# Supplementary material for: External ballistics of Pleistocene hand-thrown spears: experimental performance data and implications for human evolution
Source: Sci Rep. 2019 Jan 25;9:820. doi: 10.1038/s41598-018-37904-w (PMC6347593; doi:10.1038/s41598-018-37904-w)
Supplement: Supplementary file 6 — Supplementary Information [file 41598_2018_37904_MOESM6_ESM.pdf]

External ballistics of Pleistocene hand-thrown spears: experimental performance data and implications for human evolution

Supplementary Information

Annemieke Milks[a*], Matt Pope[a], David Parker

1. Supplementary background information

This section details background data on hand-throwing spears including those from experimental research, ethnographic sources and contemporary javelin studies.

1.1 Experimental data

Accuracy, a component of measuring 'effective distance' of early hand-thrown spears is proposed as a limitation but no known studies systematically analyse this. Churchill[1] describes a hit rate of 13.6% at a distance of 15 m by untrained throwers but details of replicas, participants, and associated velocities are not provided. Another experiment using replicas of Schöningen Spear II demonstrates that in the hands of an elite javelin athlete it can reliably hit a 200 mm wide target at a 7-8 m distance, but unfortunately 'reliability' was not quantified, and other distances were not trialled[2]. Direct comparisons with complex projectiles are difficult because studies have varying target sizes, distances, and methods for presenting results[3]. In another study[14] hand-throwing lightweight spears tipped with Mousterian points (with spears measuring between 1.8 and 2.2 m in length and weighing between 176 and 214 g), an inexperienced male thrower was capable of projecting spears at distances of over 20 m (Table S1).

Tables S1 summarises published data pertaining to hand-throwing velocities. Where possible and if not already published, estimates on KE and momentum have been calculated by the lead author (AM). Note that many velocities relate to release, and therefore calculated values for KE and momentum also relate to release, not impact and should be treated with caution. Table S2 summarises published data from experimental work mechanically projecting replicas to replicate hand-throwing of spears, again calculated KE and momentum from available data where these were not in the original publications.

Table S1. Summary of published data from previous studies of hand-throwing using human participants with relevant data on velocity, distance, and/or kinetic energy.

Thrown Object	Mass (g)	Length (mm)	Distance projected (m)	Release Velocity (m/s)	Impact Velocity (m/s)	KE (J)	P (kg*m/s)	Velocity: Estimated or Filmed	Participants / Firing mechanism	Source
**Spearthrower dart, hand-thrown**	186	2134	*	13.9 ¥	*	18.0 ¥	2.6 †	Estimated	*	[4] cited in [5]
**Spearthrower dart, hand-thrown**	182	1829	*	13.7 ¥	*	17.1 ¥	2.5 †	Estimated	*	[4] cited in [5]
**Spearthrower dart, hand-thrown**	166	1930	**mean** = 32 (n = 20)	**mean** = 18.3 (n = 20) ¥	Not known	27.8 †	3.0 †	Estimated	Two males, probably untrained	[6] Velocity estimate by [5] KE recalculated here
**Men's javelin**	800	2600 - 2700	99.72	32.3 (n = 1)	Not known	417 †	25.8 †	Filmed, 200 fps	One male javelin athlete (record setting)	[7]
**Men's javelin**	800	2600 - 2700	**range**: 83.84–89.5 **mean** = 86.46 (n = 7)	**range**: 28.1– 29.7 **mean** = 29.0 (n = 7)	Not known	**range**: 316– 353 **mean** = 336.4 (n = 7) †	**range**: 22.5 - 23.8 **mean** = 23.2 (n = 7) †	Filmed, Two synchronized SVHS Panasonic video cameras, operating at 50 fps.	Seven male javelin athletes	[8]
**Men's javelin**	800	2600 - 2700	**range**: 45.25 - 87.17	**range**: ≈18 to ≈32 (n = 57) ★	Not known	**range**: ≈130 to ≈410 †	**range**: ≈14.4 to ≈25.6 †	Filmed, video cameras, 60 fps and 200 fps (different events)	male javelin athletes (*n* = 57)	[9]
**Men's javelin**	800	2600 - 2700	**range**: 75.00 - 87.82 (excluded throws under 75 m) **mean** = 79 (n = 155)	**range**: 25.3 - 29.1 **mean** = 27.1 (n = 26)	Not known	**range**: 256 - 339 **mean** = 293.8 (n = 26) †	**range:** 20.2 - 23.3 †	'Filmed', infrared photocell gate	26 male javelin throwers	[10]
**Men's javelin**	800	2600 - 2700	**mean** = 84.6 (n = 15)	**mean** = 29.3 (n = 15)	Not known	**mean** = 343 (n = 15) †	**mean** = 23.4	Filmed,100 fps	Three male javelin athletes	[11]
**Women's javelin**	600	2200	**mean** = 55.0 (n = 13)	**mean** = 21.8 (n = 13)	Not captured	**mean** = 143 (n = 13) †	**mean** = 13.1	Filmed,100 fps	Five female javelin athletes	[11]
**Women's**	600	2200	**range**:	**range**:	Not	**range**:	**range**: 13.1 - 15.2	'Filmed', infrared	15 female javelin	[10]

**Women's javelin**	600	2200	5	Not reported	**mean** = 23.3 (n = 10)	**mean** = 163.3 (n = 10) †	**mean** = 14.0 (n = 10) †	Filmed, 200 fps	One male javelin athlete (same as for spear replica in same paper)	[2]
**Schöningen Spear II replica**	500	2300	5	Not reported	**mean** = 23.8 (n = 10)	**mean** = 140.5 (n = 10) †	**mean** = 11.9 (n = 10) †	Filmed, 200 fps	One male javelin athlete	[2]
**Throwing stick**	350 g	595	**range:** ≈60 - 110 **mean** = 77.4 (n = 27)	**range**: 23.7 - 36.2 **mean** = 29.1 (n = 27)	Not captured	**range**: 98.3 - 229.3 **mean** = 148.2 (n = 27) (not necessarily KE at impact as velocity reported is maximum, not impact)	**range**: 8.3 - 12.7 **mean** = 10.2 (n = 27) †	Bushnell Velocity Speed radar guns	Thrown by hand for distance; 27 males of Daasanach agro-pastoralists, ranging from 10 to 45 years of age, skilled in throwing	[12]
**Baseball**	144 g	N/A	10 m	maximum velocity (not release) = 27.7±3.8 n = unreported	Not captured	**mean** = 110.5; n = unreported (not necessarily KE at impact as velocity reported is maximum, not impact)	**mean** = 4.0 n = unreported (not necessarily KE at impact as velocity reported is maximum, not impact)	Sports Radal Model 3600 radar gun	Thrown by hand at a fixed target; normal unrestricted throw data; 21 male subjects ages 19-23, exclusion of 'poor throwers' as determined by accuracy and velocity	[13]
**Lithic tipped spears**	§ **range:** ≈176.3 to ≈214.4 **mean** = 189.9 (n = 8)	1800 - 2200	**range**: 5.83 - 21.93 **mean** = 14.18 (n = 69)	Not captured	Not captured	*	*	N/A	Thrown by hand for distance, untrained male participant	[14]

Notes for Supplementary Information Table S1. Estimates provided in Hughes on data from Butler's (1975) paper are not included here because they are based upon secondary data, including those from Howard 1974, which is already presented here, and the publication is concerned with spearthrower performance not hand-delivered spears. * Not possible to calculate due to missing/ unreported data. ¥ Data estimated by Hughes. † Data estimated by authors (AM), and assumes a relatively similar impact velocity to release velocity. ★Velocity data taken from regression graph as range not provided in publication. § Point and shaft mass data combined, with two grams added for hafting materials (Data kindly shared by Joseba Rios-Garaizar).

Table S2. Summary of publications with relevant data on velocity, distance and/or kinetic energy from previous experiments mechanically projecting spears to replicate hand-thrown velocities.

Thrown Object	Mass (g)	Length (mm)	Distance projected (m)	Release Velocity (m/s)	Impact Velocity (m/s)	KE (J)	P (kg*m/s)	Velocity: Estimated or Filmed	Participants / Firing mechanism	Source
**Lithic tipped spears**	**range**: 86 - 194 **mean** = 120 (n = 28)	500 (shaft, not including point)	1	Same as impact velocity due to distance	**range**: 11– 26 **mean** = 21.2 (n = 28)	**range**: 5.5 - 47.7 **mean** = 27.71 (n = 28)	**range**: 1.0 - 5.0 **mean** = 2.5 (n = 28) †	High speed video camera, 6000 fps	Mechanially projected from an air gun (2 to 6.7 bar)	[15]
**Lithic tipped spears**	**range** = 137.4 - 296.4 **mean** = 192.7 (n = 45)	Not reported	Not reported	**mean** = 25.1	Not reported, but likely to closely match release velocity	**range**: ≈43.3 to ≈97.0	**range** = ≈3.4 - ≈7.4 †	Speedtach Chronometer	Mechanically projected from custom designed device	[16]
**Lithic tipped spears**	≈266	≈570 (shaft and foreshaft, not including point)	0.93	7 - 30 **Ω**	Not reported, but likely to closely match release velocity	**range**: ≈7 to ≈120	**range**: ≈1.9 to ≈ 7.98 †	Transient-recorder and light curtains	Mechanically projected, air gun	[17,18]
**Lithic tipped spears**	≈136	Not reported	1.5	17.8 (± 1)	Not reported, but likely to closely match release velocity	≈22 †	≈2.4 †	Estimated	Mechanically projected using a calibrated crossbow	[19]

Notes for Supplementary Information Table S2. Ω Study designed to replicate both hand-thrown and spearthrower velocities, and so the upper range is suggested by the authors to represent spearthrower velocities, with brackets not defined.

Table S3. Summary of selected performance data of complex projectiles for comparative purposes

Weapon type	Mass (g) range	Velocity (m/s) range §	KE (J) range	KE (J) mean	P (kg*m/s) range	Source
**Spearthrower darts**	21.3 - 193.0	19.5 - 26.9	7.7 - 51	32.5 (n=11)	0.56 - 4.44	[5] †
**Spearthrower darts**	68 - 190	ca. 34 - 46	*	*	*	[20]
**Spearthrower darts**	44 - 195	14.8 - 38.8	11 - 87.9	*	0.99 - 5.86	[21] Table 2
**Bow/arrows**	19.3 - 49.0	30 - 65.5	13.5 - 42.5	29.9 (n=11)	0.82 - 2.0	[5] †
**Bow/arrows**	20 - 30	35.8 - 45.1	12.8 - 29.5	16.2 (n=3)	0.72 - 1.3	[21] Table 2
**Bow/arrows**	25 - 90	30 - 60	13.5 - 84.5	50.8 (n=15)	0.9 - 3.87	[22]

Notes for Supplementary Information Table S3. Data in table includes both human replicative and controlled experiments. * Cannot calculate based on data provided. § Velocities captured are often release velocities, and therefore not necessarily representative of impact velocity. † Estimates in Hughes come from a range of sources, see Hughes 1998 p 352 for detailed data. Corrections to Hughes' data have been made where calculation errors had occurred. (n.b. data also previously presented in Churchill et al. 2009, Table 1)

Table S4. Summary of a selection of five groups known to have hand-thrown spears, with associated accounts of estimated distances achieved.

Group (location)	mass (g)	distance throws (m)	aimed throws (m)	Source
**Tiwi (Melville Islands, Australia)**	1814	**range**: 31.8 - 43.7 (measured) **mean**: 36.6 (n = 9)	50	distance and mass: [23] aimed throws: [24]
**Tasmanian Aborigines (Tasmania, Australia)**	**range**: 242- 845 **mean** = 506 (n = 8)	**range**: 30 - 100	range 37 - 55	distance data: [25-27] mass data: [28]
**Aboriginal Australians, various (mainland Australia)**	*	**range**: 64-110	36-46	[29,30]
**Bari (South Sudan)**	*	46	27	[31]
**Mae Enga (Papua New Guinea)**	*	50	30	[32]

Notes for Supplementary Information Table S4. * mass data not reported. distance and aimed throws are assumed estimated unless otherwise stated.

1.2 Ethnographic data

A review of ethnographic evidence of weapon use in relation to the use of hand-delivered weaponry[33] has been particularly influential and frequently cited. Of particular relevance for this study is the oft-cited estimate from that paper for the effective distance of hand-thrown spears[18,34-41]. It proposes, on the basis of 14 groups that hand-threw spears, that they are close-range weapons, with Tasmanian Aboriginals the only group to accurately throw spears long distances[33]. With Tasmanian distance estimates included the mean throwing distance was 7.8 m (n=14), but by removing their throwing distance from the sample, a mean value of 5.7± 0.9 m (n=13) with a proposed effective range of 5-10 m was calculated[33]. Villa & Lenoir [42] questioned this range, proposing an effective distance of >20 m on the basis of further data including those from Classical sources. We provide additional examples of recent groups who threw spears with recorded and estimated distances far exceeding 5-10 m (Table S4). In particular, the case of the Tiwi needs further evaluation: although the Tiwi were included in the calculation[33] this was only on the basis of shorter estimated accuracy distances from one study[43], and it incorrectly states in the paper that the Tiwi threw light spears. Spencer[23] conducted a throwing competition of experienced Tiwi throwers, selecting a 1.84 kg spear on the basis of its average size, and the resulting distance throws averaged 36.6 m (Table S4). The mass of this spear is extremely heavy in comparison with Tasmanian throwing spears[44]. Although according to some sources[43,45] the Tiwi would approach closely when possible, they were reported elsewhere to throw their heavy spears accurately to distances of up to 50 m[24]. On mainland Australia, accuracy distances are reported in several sources to range between 36-46 m (Table S4). Although shorter throwing distances, for example of 9-14 m by the Adelaide tribe are recorded [46,47], as Cundy [48] highlights, examples of accurate throws at these distances were from groups who rarely hand-threw spears. This is a key point, because the Tiwi and Tasmanians frequently hand-threw spears and had an absence of complex projectiles [26,43,45,49]. They should therefore be considered amongst the most highly skilled throwers rather than treated as outliers. Although ethnographic accounts must always be used with caution, accuracy distances of these groups - even though exceptional - are more indicative of weapon performance when used by Pleistocene humans who did not use complex projectiles. Distances achieved with throwing spears are also likely to be dependent upon the mass of the spears, with the Tasmanians throwing relatively light spears compared with the Tiwi (Table S4), suggesting that this may also be why the Tiwi chose to approach prey closely. Outside of Australia, further examples include the Bari in South Sudan, and the Mae Enga in Papua New Guinea (Table S4). The Chabu in Ethiopia use hand-thrown spears to hunt, and although they will approach as close as 10 m if they are hunting with dogs so as not to spear them, they will often throw from much farther away [50]. While accuracy of such estimates from the literature always carry uncertainties this is equally true of the ethnographic sources forming the original estimates. A range of 5-10 m on the basis of ethnographic evidence alone is clearly underestimated, and the throwing distances of groups who regularly threw for hunting and violence must form a significant part of re-evaluating distance data.

1.3 Hand-thrown spears and javelins: release parameters, design and external ballistics

Much of what is empirically known about flight mechanics of hand-thrown spears is based on contemporary javelin use. Release velocity and angle are the most important factors in achieving distance with a thrown spear, with optimal angles in

real conditions being ca. .35 and .72 rad, corresponding with 20° to 41° [8,10,51-53]. The height of release, and hence the height of the thrower also has an impact on distance[7,52]. Body mass and height of javelin athletes are influential in distance throwing, but on average heights and body mass of modern javelin athletes are not as high as for other throwing events which throw objects with heavier mass, with technique proving a significant factor in successful javelin throwing[54] (http://www.track-stats.com/track-and-field-body-types/ accessed 21/2/2018). Olympic javelins were made of wood until the 1960s, with the Finnish birch javelins thought to represent optimal stiffness, limiting vibration in flight[55]. Today, javelins are 'distance rated', i.e. designed to match the thrower's ability with flexibility and weight distribution as key factors[55]. Lower distance throwers use more flexible javelins, rendering them more stable in flight. Elite throwers use stiffer javelins that limit vibration, as more powerful throws impart significant force, increasing vibration which in turn affects aerodynamics. Contemporary athletes spin javelins between 19 and 24 rps[51], a throwing technique that the Tasmanians may also have used[41].

The centre of gravity (point of balance) is also significant for flight mechanics. A redesign of the Olympic javelin in the 1980s aimed to limit flight distance by moving the centre of gravity 40 mm forward (now between 35% and 39% from the tip) [55]. Locating the centre of gravity forward of the middle is necessary for flight because the centre of pressure needs to act behind the centre of gravity, which helps the retarding forces orientate the spear to land point down[31]. Tapering the back of a spear keeps the centre of pressure forward, stabilising the spear in flight.

Forces including lift, drag, wind, and gravitational acceleration (a constant at 9.8 m/s^2) act upon a spear after release in turn affecting distance, flight trajectory, and yaw (angle of incidence)[48,56-58] (Supplementary Information Fig. 1). Drag occurs as a result of air resistance on the object, and is affected by an object's shape, mass, surface features and velocity: smoother surfaces, smaller objects, streamlined shapes, lower velocities and higher mass all reduce drag. Although a javelin and large spear have a large surface area, their streamlined shape, relatively high mass and velocities mean that the drag coefficient is not particularly high compared with other thrown projectiles. Therefore at lower velocities, projectiles with a higher mass will be less affected by drag. Javelin/spear design also influences drag and lift. Javelins and spears with larger diameters increase lift [58]. If a spear's long axis is perpendicular to the flight trajectory, as would occur in a vacuum, maximum drag and stalling occur [31], but the combination of forces acting on a spear in real conditions typically orients a spear at a tangential angle to its flight path (Supplementary Information Fig. 2).

As it requires greater energy to propel objects with a higher mass at an equal velocity[59], throwing velocities decrease with increases in mass. The design of spears reflects a balancing of multiple features, with the design of the Olympic javelin reflecting a contemporary mathematically-based understanding of projectile behaviour. As one of the most aerodynamic of sporting projectiles, it is optimised for distance throwing and is matched to the athlete and field conditions.

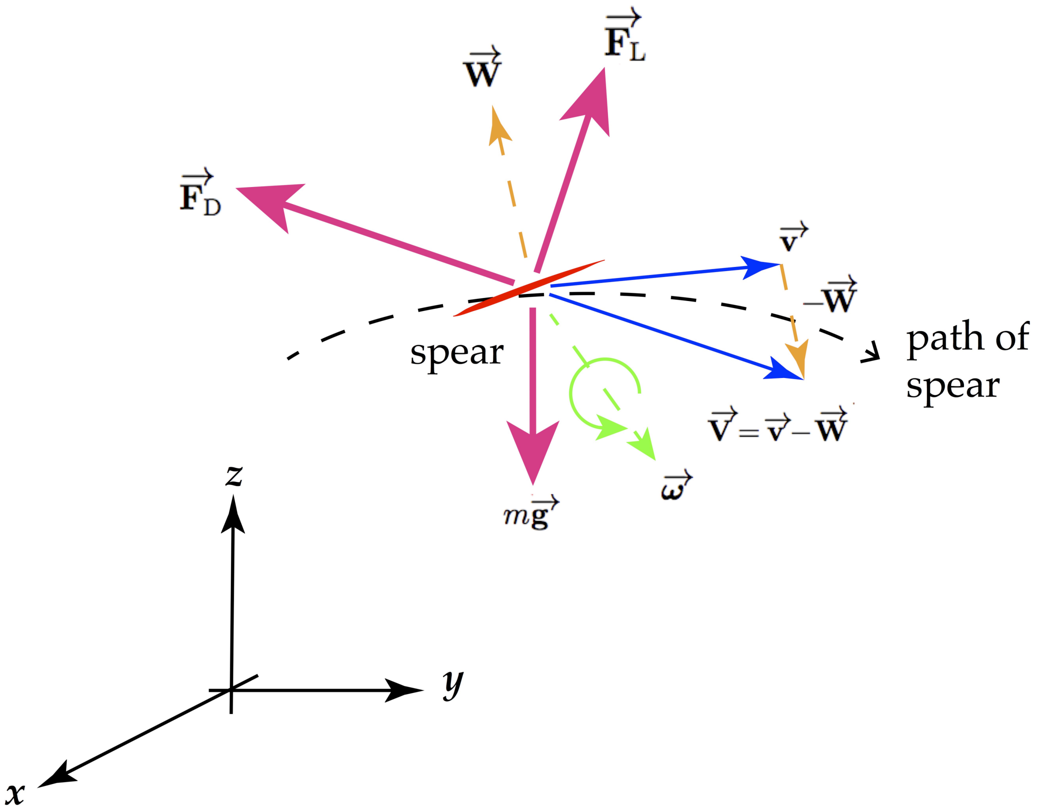

Figure 1. Schematic drawing showing forces acting on a spear during flight in field conditions. F_D represents the drag force, F_L the lift force, and *m*g the gravitational force. W represents the wind velocity and v the projectile velocity in relation to the coordinates. V = v – W represents the projectile velocity relative to the air, while $\vec{\omega}$ is the projectile angular velocity. Redrawn and recaptioned by A. Milks after [60].

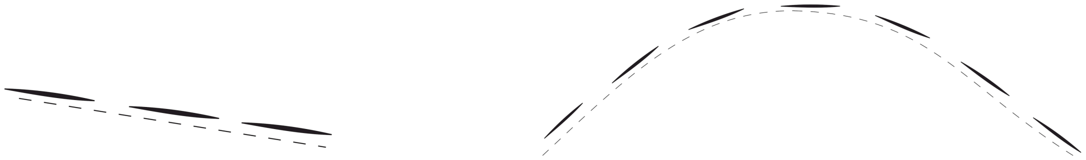

Figure 2. Schematic drawings of flight trajectories (dotted line). Left, flat flight trajectory, right: parabolic flight trajectory. Both illustrations reflect an optimal tangential orientation of the spear to trajectory, landing point first. Drawn by A. Milks.

Wind conditions can both negatively and positively affect a spear's flight behaviour and distance[7,57,58,61]. Tailwinds can increase the horizontal velocity vector, as well as affecting lift during flight. The following equation (1) (after [61]) demonstrates how wind velocity vector is affected by wind speed:

Equation 1. Wind velocity vector

$$T_0 = W - V_0$$
$$W = [\lambda \cos a, \lambda \sin a]^T$$

Where:
T_0 = initial relative air velocity vector
V_0 = release velocity vector
W = wind velocity vector
λ = wind speed
a = horizontal angle
T = relative air velocity vector

The effects of tailwinds and headwinds vary depending upon design of a thrown spear due to differences in the relationship between the centre of mass and centre of pressure as well as in the design of the proximal section of the spear[61]. Wind conditions can be somewhat moderated by release parameters including release angle (relative to the ground) and angle of incidence (yaw), and contemporary javelins are variably designed to maximise different wind conditions.

Gravitational acceleration also affects the impact velocity, and in part explains instances in the experiment where there are increases in velocity in the experiment from release to impact. The simple trajectory model of a parabola, stating that speed is the same at landing as it is at launch assumes that the launch height and landing height are the same, which is untrue for most projectiles in sports, including the javelin/spear[58]. The simple model also ignores lift, drag and wind conditions . The spears were released at a greater height than the height they impacted the target. Velocities at release were calculated at >1.5 m above the ground, as all the participants were >1.78 m tall and release took place between shoulder height and above the head depending on the throw while impacts were near ground level. In a vacuum, a flat throw that gained ca. 1.5 m in height between release and impact, vertical velocity gained would be 5.4 m/s, but the effects of drag mitigate this increase. For parabolic throws this is different because the gain in velocity is not linear but quadratic. For example, ignoring drag, impact velocity from an object falling from a 9 m height would be 13.2 m/s, while impact velocity of an object falling from a 10 m height would be 14.0 m/s. An increase from 9 to 10 m height results in a 0.8 m/s gain in vertical velocity. Therefore overall slight increases in velocity between release and impact is to be expected. If the target is at the same or similar height as the thrower, such increases would be negated. The equations (2) for calculating velocity are:

Equation 2.

$$t = \sqrt{[d/(0.5g)]}$$
$$v = gt$$

Where:
t = time
d = distance
g = gravitational acceleration (9.81 m/s^2)
v = velocity

1.4 Biomechanics

The modern javelin grip, called ‘Greek’ or ‘orthodox’, is a single-handed underhand hold at the centre of the spear, using the dominant hand with small variations in finger positioning (Supplementary Information Fig. 3)[55,58]. In ‘free-style’ events javelin athletes used to use a variety of throwing techniques, including holding the javelin at the rear and propelling it[55]. Current rules are much more restrictive in terms of technique, but it is important to recognise that there are multiple throwing techniques that may maximise different release conditions.

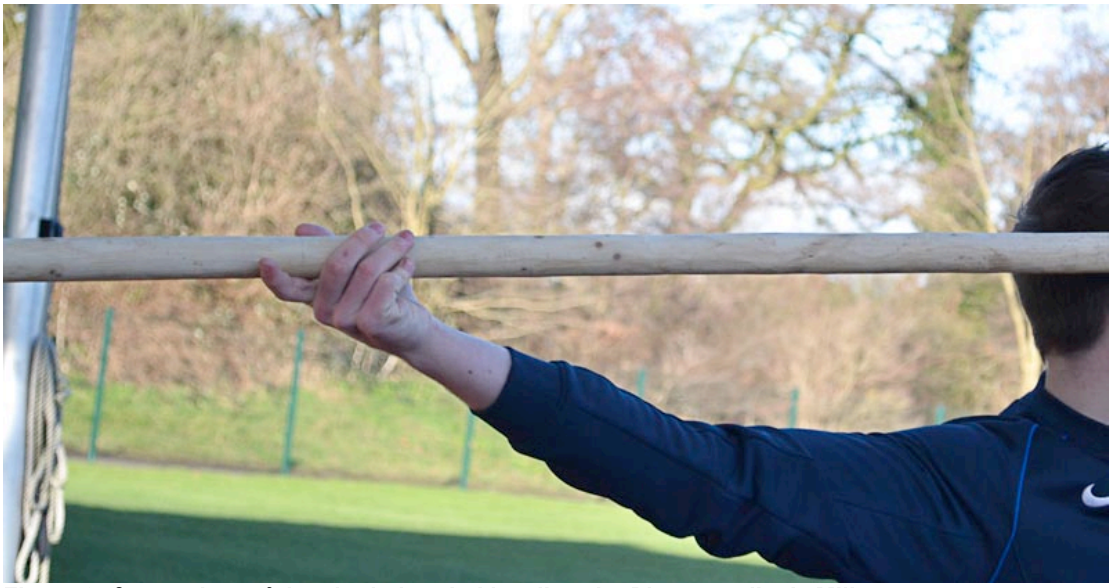

Figure 3. One variant of the single-handed javelin grip. Photo credit: A. Milks.

2. Supplementary methods

This section provides further details on the methods for the experimental work.

2.1 Spear replicas

The trees used for replicas were grown in warm conditions, so trees with a circumference larger than necessary for the finished product were used enabling the use of higher density heartwood. This facilitated a replication of the density and therefore mass of the original, as Schöningen Spear II was manufactured from dense slow-grown spruce[62,63]. The distal ends of the spears were created from the bases of the trees with tips offset from the medullary canal, as they were at Schöningen[64]. Four replicas were created, and the two with the most similar masses and points of balance to each other within the sample were selected for use (Table S5). Replica 1 broke after on the final aimed throw (25 m), and replica 2 was used for the subsequent 10 metre throws with the vertical hay bale, and for the distance throws.

Table S5. Measurement data of the spear replicas selected for use

Replica	Length	Ø at 10 mm	Ø at 50 mm	Ø at 800 mm	Ø at 1150 mm (midpoint)	Ø at 1530 mm	Mass (g)	P.O.B. (mm)
**1**	2300	6	19	35	33	37	760	1091 (47%)
**2**	2300	7	17	37	34	34	800	1084 (47%)

*** As measured from distal point of spear. Length, Diameter (Ø), and Point of Balance (P.O.B.). measurements in mm, mass in grams. P.O.B. measured from distal tip.**

2.2 Data analysis

Due to the dynamic nature of the spear throwing and issues with the availability of light when filming outside, videos had some variation in what the filming captured. For release videos, estimated velocities were calculated from the frame that the spear left the hand, in order to ensure that release velocities of the spear were calculated, rather than velocity of the throwing arm. For release videos, the final frame was as close to the moment when the point or rear of the spear (depending upon which was used in calculations) reached the edge of the video frame. A further variable calculated in video analysis was angle (radians), measured as the difference from a vertical line on the video frame, spanning from the first to the last frame for which velocity measurements are calculated (Supplementary Information Fig. 4). For impact videos, estimated velocity was calculated from 20 frames prior to the frame where impact was determined visually to have occurred. This distance ensures that velocity captured represents impact velocity. A sample analysis video (Supplementary video 5) is included to demonstrate the analysis process for an impact video. Edge detection algorithms were applied when necessary to clearly define the objects for measurement. As velocity is based upon a distance measurement, the maximum error for the velocities was calculated by setting the calibration scale to the known measurement of the spear, and then measuring another known value in the video. The maximum error for each velocity is 4% of the value. For a velocity of 16 m/s the error range is 16 ± 0.64 m/s. Parallax may account for additional small error values, as the camera was set up at a right angle to the spear trajectory as it approached the target, and spear angles (yaw and impact) in field conditions are also variable.

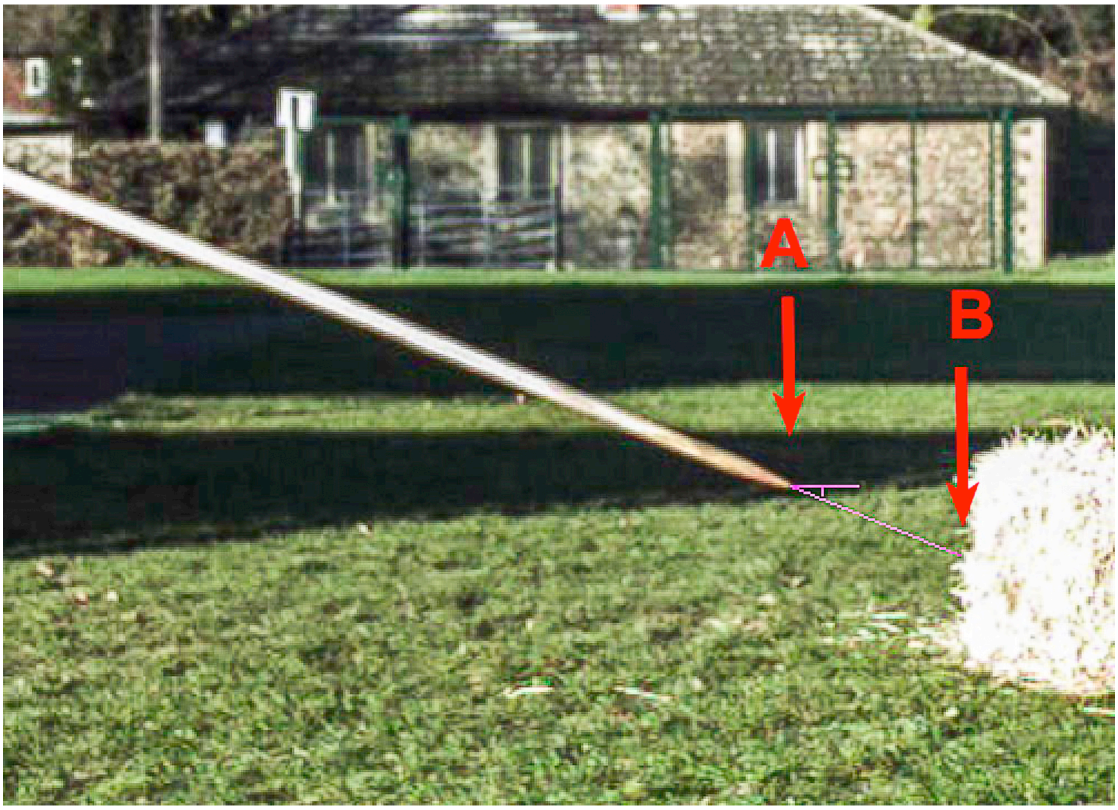

Figure 4. Throwing video analysis. The pink line represents the distance measured in the video frame. The angle represents the angle measurements automatically calculated by Cine Viewer (expressed as radians). 'A' represents the starting point of the measurement in the video, 20 frames before impact and 'B' the end point of measurement taken. Photo credit: A. Milks.

It was not possible to capture impact velocities for three of the distance throws, and three further videos were unsuitable for analysis due to problems with clearly identifying the entire spear in the frame. This left six HSVs from which to analyse velocities from distance throws, though several videos didn't capture moment of impact with the ground, and so velocities captured in those cases are only to be considered as estimates for impact velocities.

2.3 Kinetic energy and momentum

Both kinetic energy and momentum are significant factors in the determining wounding potential of a weapon. Kinetic energy (KE), the energy an object has due to its motion, is an important variable for understanding the effects of impact of projectiles. The equation (3) for KE (J) is:

Equation 3. Kinetic energy.

$$KE = \frac{1}{2} m * v^2$$

Where:
m = mass
v = velocity

Increases in velocity are more influential on KE than mass. The influence of mass on KE is linear, while the influence of velocity on KE is quadratic. However the *relationship* between the influence of mass vs. the influence of velocity on KE is exponential. Supplementary Information Fig. 5 shows a theoretical model. The blue triangles show increase in mass while velocity remains constant, while the green circles demonstrate increase in velocity while mass remains constant, with increases in each case by a factor of 1.5. The calculations underpinning the plot demonstrate that it is the difference of the differences between the increases in mass and velocity that are exponential in nature, i.e. the change is not a constant value. Increases in mass at lower velocities such as those in spear throwing are more influential on KE than increases in mass at higher velocities.

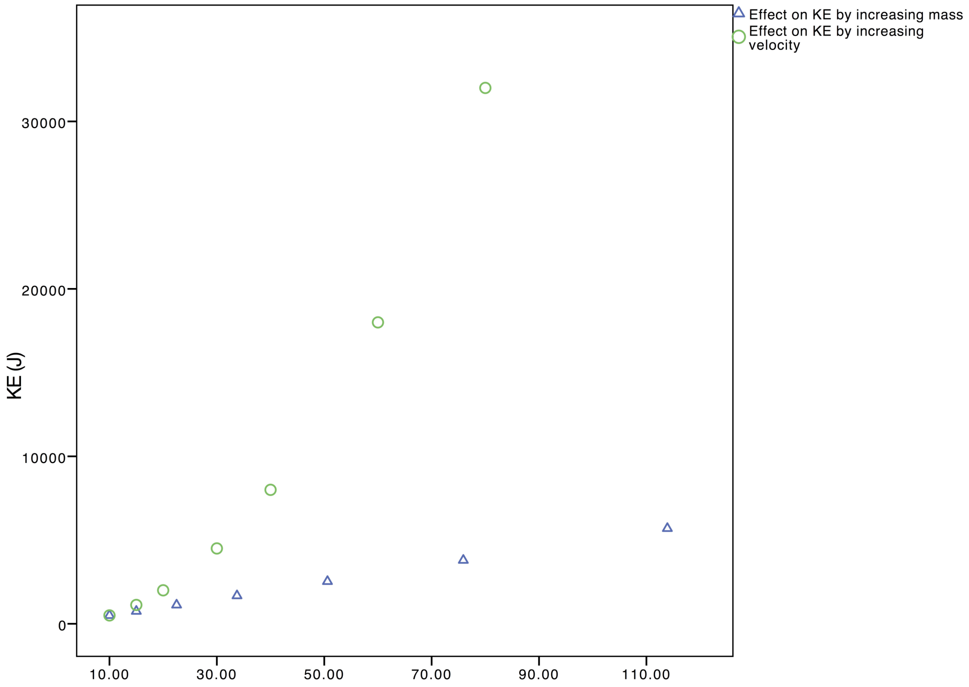

Figure 5. Theoretical scatterplot comparing effects of increases of velocity (green circles) and mass (blue triangles) on KE (y axis). Values begin at 10 (m/s and kg) and increase by factors of 1.5.

Momentum (*p*) is a property of a moving body resulting from its mass and motion and its conservation is embodied in Newton's First Law stating that an object at rest stays at rest and an object in motion stays in motion unless a force acts upon it. It is equal to the product of mass and velocity, and is related to KE in that the variables are the same, but unlike KE increases are linear. Like KE it is a vector, as momentum has direction as well as speed. The equation for momentum (SI unit: kg*m/s) is:

Equation 4. Momentum

$$p = m * v$$

Where:
m = mass
v = velocity

As prehistoric projectile studies often reference or produce data on momentum [21,65-68]

these values are also provided in this paper.

3. Supplementary results

3.1 Participant data

Table S6. Participant personal data

Participant	Age	Height (m)	Weight (kg)	Personal Best (m)	Yrs throwing experience	Dominant Hand
**1**	19	1.83	83	57.27	5	R
**2**	34	1.89	93	78.33	24	R
**3**	19	1.78	78	60.62	9	R
**4**	19	1.73	90	39.73	5	L
**5**	18	1.78	65	33	1	R
**6**	18	1.88	81	51.42	4	R

Table S7. Hit and Miss data by participant

Participant	Years throwing	Total number aimed throws	Number of hits	% of total resulting in a hit
**1**	5	18	5	28
**2**	24	18	4	22
**3**	9	18	4	22
**4**	5	18	6	33
**5**	1	18	2	11
**6**	4	18	4	22

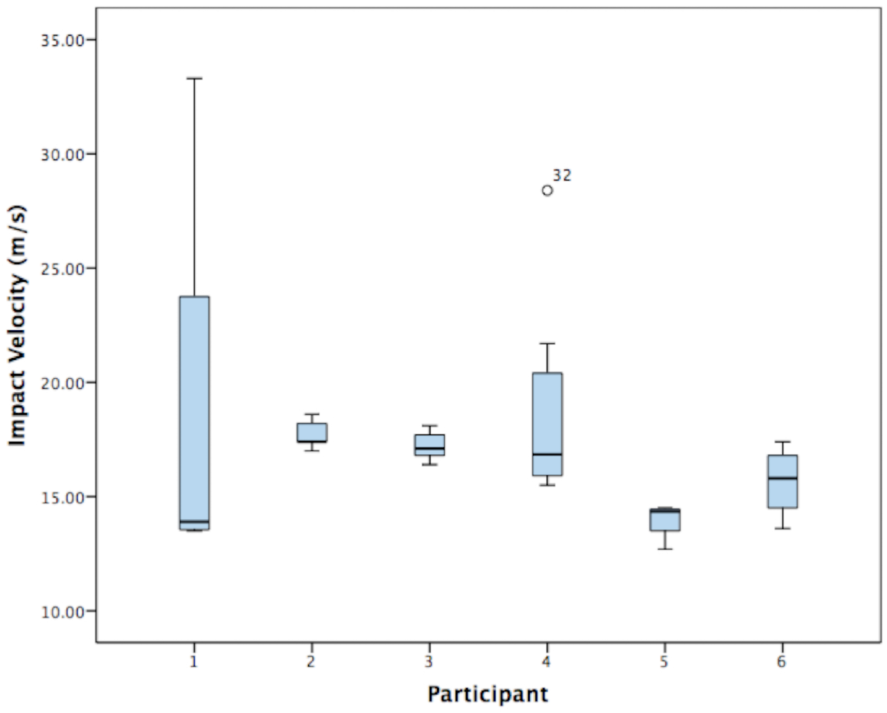

Figure 6. Boxplot of impact velocities by participant.

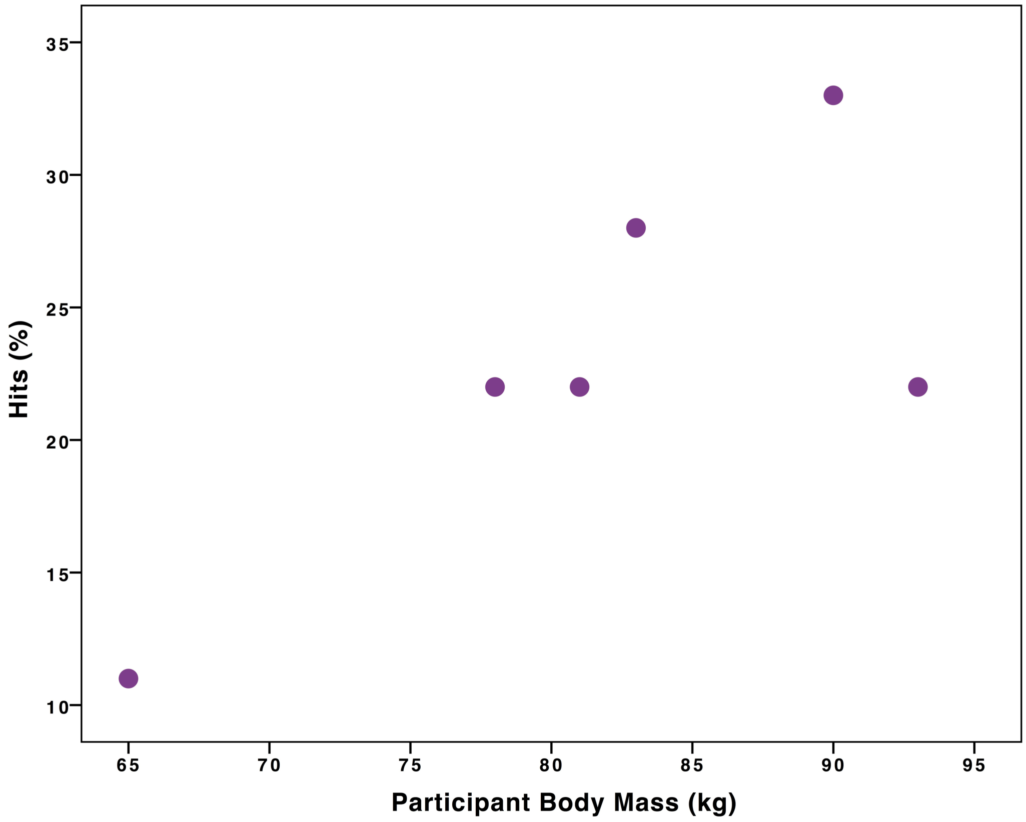

Figure 7. Scatterplot of participant body mass and hit rate.

3.2 Release velocity

A histogram of release velocities from the experiment showed a multimodal distribution (Supplementary Information Fig. 8). A Shapiro-Wilk test (p = 0.999) along with a visual inspection of the Q-Q plot confirms a normal distribution.

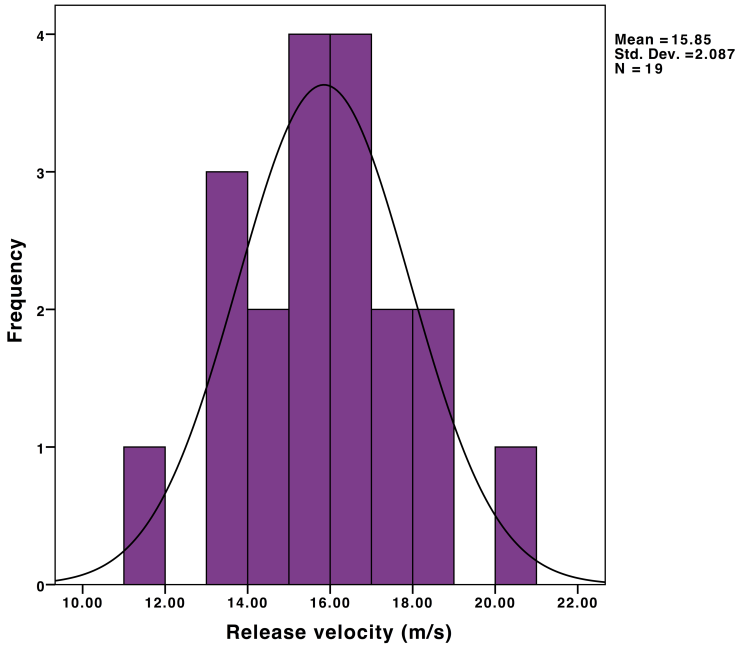

Figure 8. Histogram of release velocities.

3.3 Impact velocity

A histogram of impact velocities (Supplementary Information Fig. 9) demonstrates the presence of outliers in the dataset, and a non-normal distribution, confirmed by a visual inspection of the Q-Q plot and a Shapiro Wilk test (p=0.000). Removing the two outliers creates a normal distribution with a Shapiro Wilk test confirming this (p=0.472).

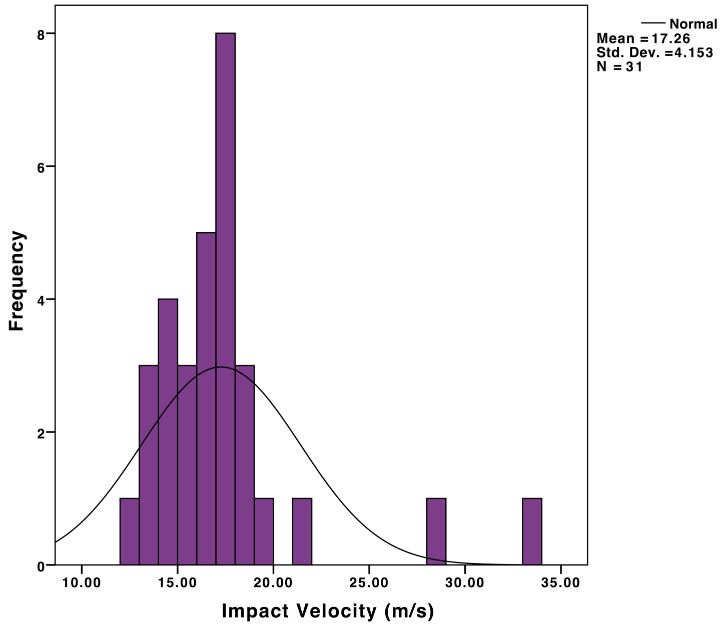

Figure 9. Histogram of impact velocities, combined target and distance throws

3.4 Supplementary images for flight trajectories and angles at release and impact

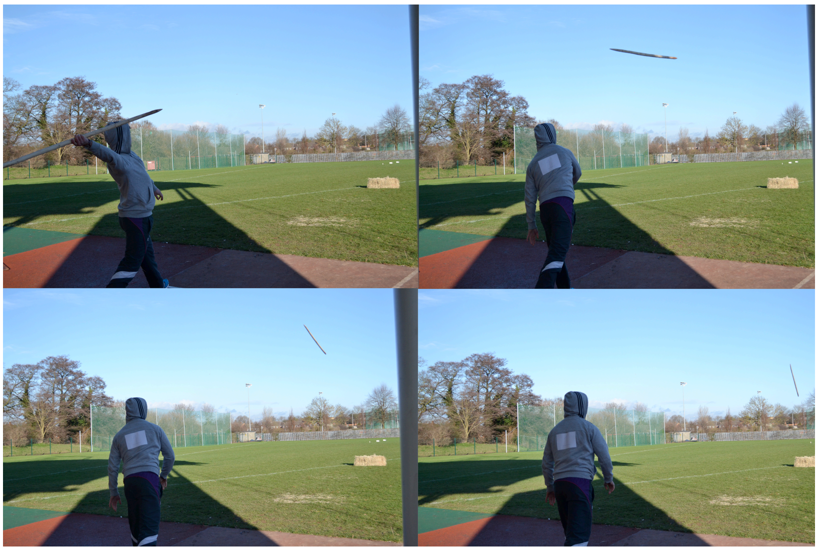

Figure 10. An example of a target throw at 15 m, starting top left to right, then bottom left to right. Photo credit: A. Milks.

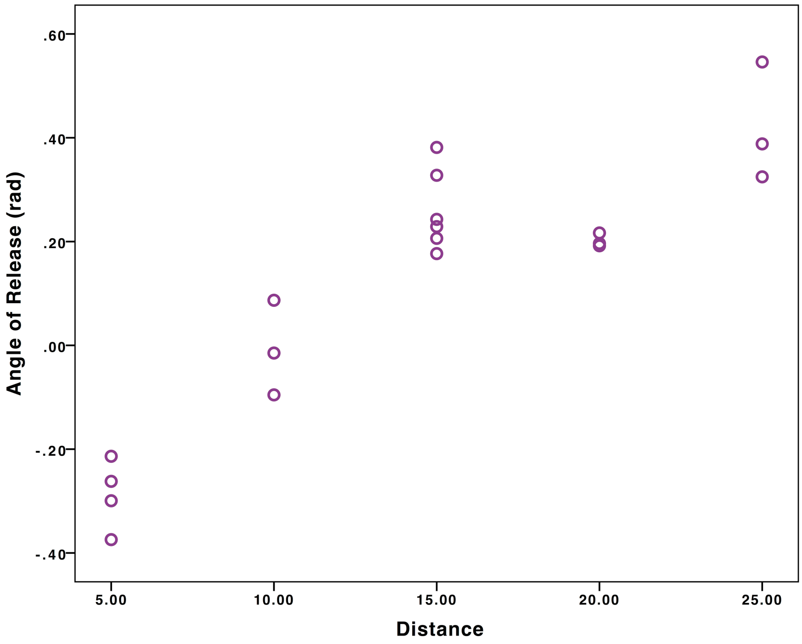

Figure 11. Impact angle (rad) measured in release video footage, separated by distance of targets.

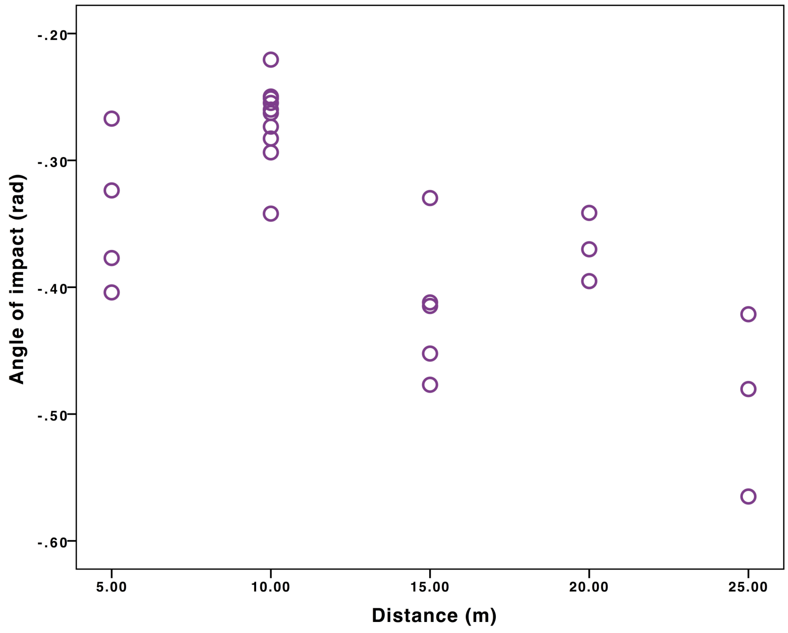

Figure 12. Angle (rad) measured in impact video footage (y axis), separated by distance of targets (x axis).

References

1. Churchill, S. E. *Thin on the ground: Neandertal biology, archeology, and ecology*. 1–256 (Wiley Blackwell, 2014).
2. Rieder, H. Erprobung der Holzspeere von Schöningen (400000 Jahre) und Folgerungen daraus. In *Fruhe Menschen in Mittel Europa: Chronologie, Kultur, Umwelt* (eds. Wagner, G. A. & Mania, D.) 91–98 (Shaker, 2001).
3. Hutchings, W. K. & Brüchert, L. W. Spearthrower performance: ethnographic and experimental research. *Antiquity* **71,** 890–897 (1997).
4. Van Buren, G. E. *Arrowheads and projectile points*. (Arrowhead Publishing Co., 1974).
5. Hughes, S. S. Getting to the point: evolutionary change in prehistoric weaponry. *J Archaeol. Method Theory* **5,** 345–408 (1998).
6. Howard, C. D. The atlatl: function and performance. *American Antiquity* 102–104 (1974).
7. Gregor, R. J. & Pink, M. Biomechanical analysis of a world record javelin throw: A Case Study. *International J. Sport Bio.* **1,** 73–77 (1985).
8. Campos, J., Brizuela, G. & Ramón, V. Three-dimensional kinematic analysis of elite javelin throwers at the 1999 IAAF World Championships in Athletics. *New Stud. Athl.* **19,** 47–57 (2004).
9. Murakami, M. *et al.* Biomechanical analysis of the javelin at the 2005 IAAF World Championships in Athletics. *New Stud. Athl.* **21,** 67 (2006).
10. Viitasalo, J., Mononen, H. & Norvapalo, K. Release parameters at the foul line and the official result in javelin throwing. *Sports Biomech.* **2,** 15–34 (2003).
11. Rich, R., Gregor, R., Whiting, W. & McCoy, R. Kinematic analysis of elite javelin throwers. *Track and Field Quarterly Review* **86,** 35–38 (1986).
12. Roach, N. T. & Richmond, B. G. Clavicle length, throwing performance

and the reconstruction of the Homo erectus shoulder. *J. Hum. Evol.* **80,** 107–113 (2015).
13. Roach, N. T. & Lieberman, D. E. Upper body contributions to power generation during rapid, overhand throwing in humans. *J. Exp. Biol.* **217,** 2139–2149 (2014).
14. Rios-Garaizar, J. Experimental and archeological observations of northern Iberian peninsula Middle Paleolithic Mousterian point assemblages. Testing the Potential Use of Throwing Spears Among Neanderthals. In *Multidisciplinary Approaches to the Study of Stone Age Weaponry* (eds. Iovita, R. & Sano, K.) 213–225 (Springer, 2016).
15. Milks, A. Spear throwing in the European early Upper Palaeolithic. MSc dissertation, UCL (2010).
16. Hutchings, W. K. Measuring use-related fracture velocity in lithic armatures to identify spears, javelins, darts, and arrows. *J. Archaeol. Sci.* **38,** 1737–1746 (2011).
17. Iovita, R., Schönekeß, H., Gaudzinski-Windheuser, S. & Jäger, F. Projectile impact fractures and launching mechanisms: results of a controlled ballistic experiment using replica Levallois points. *J. Archaeol. Sci.* **48,** 73–83 (2014).
18. Iovita, R., Schönekeß, H., Gaudzinski-Windheuser, S. & Jäger, F. Identifying weapon delivery systems using macrofracture analysis and fracture propagation velocity: a controlled experiment. In *Multidisciplinary Approaches to the Study of Stone Age Weaponry* (eds. Iovita, R. & Sano, K.) 13–28 (Springer, 2016).
19. Sano, K. & Oba, M. Backed point experiments for identifying mechanically-delivered armatures. *J. Archaeol. Sci.* **63,** 13–23 (2015).
20. Tolley, R. & Barnes, I. Reinventing the atlatl. *J. of Steward Anth Soc* **10,** 161–179 (1979).
21. Whittaker, J. C., Pettigrew, D. B. & Grohsmeyer, R. J. Atlatl dart velocity: accurate measurements and implications for Paleoindian and archaic archaeology. *PaleoAmerica* **3,** 161–181 (2017).
22. Bergman, C. A., McEwen, E. & Miller, R. Experimental archery: projectile velocities and comparison of bow performances. *Antiquity* **62,** 658–670 (1988).
23. Spencer, W. B. *Native tribes of the Northern territory of Australia*. (Macmillan and Co., 1914).
24. Morris, J. Relationship between the British and the Tiwi in the vicinity of Port Dundas, Melville Island. *Historical Society of the Northern Territory* (1964).
25. Lloyd, G. T. *Thirty-three years in Tasmania and Victoria: being the actual Experience of the author, interspersed with historic jottings, narratives, and counsel to emigrants.* (Houlsten and Wright, 1862).
26. Roth, H. L. *The aborigines of Tasmania*. (Kegan Paul, Trench, Trübner & Co, 1890).
27. Robinson, G. A. *Friendly mission: the Tasmanian journals and papers of George Augustus Robinson 1829-1834*. (Tasmanian Historical Society Research Association, 1966).
28. Milks, A. Lethal Threshold: The evolutionary implications of Middle Pleistocene wooden spears. PhD thesis, UCL (2018). http://discovery.ucl.ac.uk/10045809/
29. Giles, E. *Australia twice traversed*. (Libraries Board of South Australia, 1889).
30. Christison, R. & Edge-Partington, J. 19. Notes on the weapons of the Dalleburra Tribe, Queensland, lately presented to the British Museum by Mr. Robert Christison. *Man* **3,** 37 (1903).

31. Baker, S. W. *Ismailia*. **1,** (Macmillan, 1874).
32. Meggitt, M. J. *Blood is their argument: Warfare among the Mae Enga tribesmen of the New Guinea*. (McGraw-Hill Humanities Social, 1977).
33. Churchill, S. E. Weapon technology, prey size selection, and hunting methods in modern hunter-gatherers: implications for hunting in the Palaeolithic and Mesolithic. *Arch. Papers of the Am. Anth. Assoc.* **4,** 11–24 (1993).
34. Kennedy, K. A. R. Slings and arrows of predaceous fortune: Asian evidence of prehistoric spear use. *Evol. Anthropol.* **13,** 127–131 (2004).
35. Shea, J. The origins of lithic projectile point technology: evidence from Africa, the Levant, and Europe. *J. Archaeol. Sci.* **33,** 823–846 (2006).
36. Lieberman, D. E., Bramble, D. M., Raichlen, D. A. & Shea, J. J. The evolution of endurance running and the tyranny of ethnography: A reply to Pickering and Bunn (2007). *J. Human Evol.* **53,** 439–442 (2007).
37. Shea, J. & Sisk, M. Complex projectile technology and Homo sapiens dispersal into western Eurasia. *PA.* **2010,** 100–122 (2010).
38. Wilkins, J., Schoville, B. J. & Brown, K. S. An experimental investigation of the functional hypothesis and evolutionary advantage of stone-tipped spears. *PLoS ONE* **9,** e104514 (2014).
39. Sano, K., Denda, Y. & Oba, M. Experiments in fracture patterns and impact velocity with replica hunting weapons from Japan. In *Multidisciplinary Approaches to the Study of Stone Age Weaponry* (eds. Iovita, R. & Sano, K.) 29–46 (Springer, 2016).
40. Gaudzinski-Windheuser, S. Hunting lesions in Pleistocene and early Holocene European bone assemblages and their implications for our knowledge on the use and timing of lithic projectile technology. In *Multidisciplinary Approaches to the Study of Stone Age Weaponry* (eds. Iovita, R. & Sano, K.) **24,** 77–100 (Springer Netherlands, 2016).
41. Wilkins, J. & Schoville, B. Edge damage on 500-Thousand-Year-Old spear tips from Kathu Pan 1, South Africa: the combined effects of spear use and taphonomic processes. In *Multidisciplinary Approaches to the Study of Stone Age Weaponry* (eds. Iovita, R. & Sano, K.) 101–117 (Springer, 2016).
42. Villa, P. & Lenoir, M. Hunting and hunting weapons of the Lower and Middle Paleolithic of Europe. In *The Evolution of Hominin Diets* (eds. Hublin, J.-J., Richards, M. P. & Richards, M. P.) 59–85 (Springer, 2009).
43. Goodale, J. C. *Tiwi Wives: a study of the women of Melville Island, North Australia*. (Prospect Heights Ill, 1971).
44. Noetling, F. Notes on the hunting sticks (lughkana), spears (perenna), and baskets (tughbrana) of the Tasmanian Aborigines. *Papers and Proceedings of the Royal Society of Tasmania* 64–98 (1911).
45. Hart, C. W. M. & Pilling, A. R. *The Tiwi of North Australia*. (Holt, Reinhart & Winston, 1960).
46. Eyre, E. J. *Journals of expeditions of discovery into Central Australia and pverland from Adelaide to King George's Sound.* **2,** (Boone, 1845).
47. Tindale, N. B. Natives of Groote Eylandt and the West Coast of the Gulf of Carpentaria. Records of the South. *Australian Museum* **3,** 61–134 (1925).
48. Cundy, B. J. *Formal variation in Australian spear and spearthrower technology*. **546,** (BAR International Series, 1989).
49. Hiatt, B. The food quest and the economy of the Tasmanian Aborigines (Continued). *Oceania* **38,** 190–219 (1968).
50. Dira, S. J. & Hewlett, B. S. Learning to spear hunt among Ethiopian Chabu adolescent hunter-gatherers. In *Social Learning and Innovation in Contemporary Hunter-Gatherers* **15,** 71–81 (Springer Japan, 2016).

51. Terauds, J. Javelin release characteristics. *Track Technique* **61,** 1945 (1975).
52. Miller, D. I. & Munro, C. F. Javelin position and velocity patterns during final foot plant preceding release. *J. Human Move. Studies* **9,** 1–20 (1983).
53. Komi, P. & Mero, P. V. Biomechanical analysis of Olympic javelin throwers. *International J. Sport Biomech.* **1,** 139–150 (1985).
54. Čoh, M., Milanović, D. & Embersić, D. Anthropometric characteristics of elite junior male and female javelin throwers. *Coll. Antropol.* **26 Suppl,** 77–83 (2002).
55. Johnson, C. *Javelin Throwing*. (British Amateur Athletic Board, 1987).
56. Cotterell, B. & Kamminga, J. *Mechanics of Pre-industrial technology: an introduction to the mechanics of ancient and traditional material culture.* (Cambridge University Press, 1989).
57. Bartlett, R. M. The aerodynamics of javelin flight - a re-evaluation. The International Symposium on Biomechanics in Sports (eds. Tsarouchas, L., Terauds, J., Gowitzke, B. A. & Holt, L. E.) 71–87 (1987).
58. White, C. *Projectile dynamics in sport: principles and applications*. (Routledge, 2010).
59. Toyoshima, S. & Mitsumasa, M. Force-velocity relation in throwing. *Research quarterly. American association for health, physical education and recreation* **44,** 86–95 (1973).
60. Robinson, G. & Robinson, I. The motion of an arbitrarily rotating spherical projectile and its application to ball games. *Phys. Scr.* **88,** 018101 (2013).
61. Chiu, C. H. Discovering optimal release conditions for the javelin world record holders by using computer simulation. *International J. Sport and Exercise Sci.* **1(2),** 41-50 (2009).
62. Thieme, H. Lower Palaeolithic hunting spears from Germany. *Nature* **385,** 807–810 (1997).
63. Schoch, W. H., Bigga, G., Böhner, U., Richter, P. & Terberger, T. New insights on the wooden weapons from the Paleolithic site of Schöningen. *J. Human Evol.* **89,** 214–225 (2015).
64. Thieme, H. Lower Palaeloithic throwing spears and other wooden implements from Schöningen, Germany. In *Hominid evolution: lifestyles and survival strategies* (ed. Ullrich, H.) 383–395 (Gelsenkirchen Germany : Edition Archaea, 1999).
65. Churchill, S. E., Franciscus, R., McKean-Peraza, H. A., Daniel, J. & Warren, B. R. Shanidar 3 Neandertal rib puncture wound and paleolithic weaponry. *J. Human Evol.* **57,** 163–178 (2009).
66. Tomka, S. A. The adoption of the bow and arrow: a model based on experimental performance characteristics. *American Antiquity* **78,** 553–569 (2013).
67. Schoville, B. & Brown, K. S. Comparing lithic assemblage edge damage distributions: examples from the Late Pleistocene and preliminary experimental results. *Explorations in Anthropology* **10,** 34–49 (2010).
68. Schoville, B. J., Wilkins, J., Ritzman, T., Oestmo, S. & Brown, K. S. The performance of heat-treated silcrete backed pieces in actualistic and controlled complex projectile experiments. *J. Archaeol. Sci. Rep.* **14,** 302–317 (2017).
